# The Integrated Function of the Lateral Hypothalamus in Energy Homeostasis

**DOI:** 10.3390/cells14141042

**Published:** 2025-07-08

**Authors:** Xiangtong Chen, Yutong Wang, Su Fu, You Wan, Jian Mao, Kun Cui, Hong Jiang

**Affiliations:** 1 Beijing Life Science Academy, Beijing 102209, China; 2 Department of Neurobiology, Neuroscience Research Institute, School of Basic Medical Sciences, Peking University, Beijing 100871, China; 3 Key Laboratory for Neuroscience, Ministry of Education/National Health Commission, Peking University, Beijing 100871, China

**Keywords:** lateral hypothalamus, energy homeostasis, eating behavior, single cell sequence

## Abstract

The lateral hypothalamic area (LHA) serves as a central integrative hub for the regulation of energy homeostasis and motivational behaviors, including feeding and arousal. Recent advances in single-cell transcriptomics have revealed remarkable molecular heterogeneity within the LHA, identifying more than 30 distinct neuronal subtypes, such as GABAergic (LHA^Vgat^), glutamatergic (LHA^Vglut2^), orexin, melanin-concentrating hormone (MCH), and leptin receptor-expressing (LHA^Lepr^) neurons. These neuronal populations sense peripheral metabolic signals—such as leptin, insulin, and glucose—both directly and indirectly, and they coordinate appropriate physiological and behavioral responses through local circuits and reciprocal connections with other hypothalamic nuclei. Furthermore, the LHA interfaces with extrahypothalamic regions, including the ventral tegmental area (VTA), nucleus accumbens (NAc), and lateral habenula (LHb), thereby linking metabolic state to reward processing and behavioral prioritization. In this review, we summarize and integrate recent molecular and functional findings to present a comprehensive view of the LHA as a dynamic, multifunctional center in the central regulation of metabolism. A deeper understanding of these mechanisms may offer new therapeutic avenues for addressing obesity and related metabolic disorders.

## 1. Brief History of Identifying the Lateral Hypothalamus

The term hypothalamus was coined in 1893 by Wilhelm His, who was an outstanding anatomist and embryologist and a pioneer of modern neuroscience [1]. Despite the difficulty in accurately determining the first paper referring to the hypothalamus, there seems to be a high degree of consensus regarding the fundamental and integrative function of this region [2]. The earliest definition of hypothalamus nuclei can be originated from G.T. Ziehen (1901) and S. Rámon y Cajal (1904), whose work opened systematic advances in identifying and partitioning this small but “chaotic” tissue [2]. Thanks to the invention of the Horsley–Clarke stereotaxic instrument, scientists were given an efficient tool to manipulate the very specific areas of hypothalamus by electrical lesions and stimulation [3]. This marked the inception of the intriguing quest to understand the lateral hypothalamus (LHA) and delve into its complex neurophysiological functions. In 1951, Anand and Brobeck serendipitously found that bilateral destruction of the lateral hypothalamus in rats is followed by a complete absence of eating [4,5]. Adipsia syndrome was next observed and can be rescued by hypertonic saline injection [6]. Encouraged by this inspiring discovery in LHA-lesioned rats, numerous scientists further explored the consequences and investigated the structural and functional characteristics of the LHA, which was called the “feeding center” at that time.

The name “lateral hypothalamus” seems to be insufficiently defined, and that turns out to be true. Dating back to several decades ago when the study of hypothalamus was just thriving, the LHA was roughly divided into three rostro-caudal zones—anterior (aLHA), tuberal (tLHA), and posterior (pLHA)—based on their spatial location, covering approximately the half hypothalamus region. Many researchers were focused on the sub regionalization of the LHA based on differences in the morphological and functional characteristics and neural projections of different cells in this region [7,8]. Some classic nuclei in the hypothalamus, such as the lateral tuberal nucleus (TUM) and supraoptic nucleus (SON), were even classified as being part of the LHA at that time, which led to LHA classification being imprecise and lacking clarity. Even until recently, the LHA was poorly defined and limited to a smaller anatomical volume [9]. The groups with “prominent cellular clusters” such as SON and TUM have been excluded from the LHA and investigated independently [7]. The latest definition is more specific, referring to those cells as “loosely organized cellular masses”, implying that the historically designated remnants of the LHA possess a highly integrated and complex composition and cytoarchitecture that is still not fully understood.

The discoveries of two neuropeptides located in the LHA, melanin-concentrating hormone (MCH) and orexin (also called hypocretins), is another milestone in the history of the LHA [10,11,12]. But, still, many neurons are poorly understood, restricted by the limits of technology. It was not until the application of single-cell (scRNA) sequencing technology that the complete picture of the molecular architecture of the LHA was shown. Neurons in the LHA can be classified into more than 30 subtypes based on gene markers. Despite ongoing refinements in its anatomical definition and cell-type components, one constant since the discovery of the LHA has been its pivotal role in the regulation of energy homeostasis. In this review, we summarize and discuss how various cell types within the LHA detect internal and external energy-related signals, integrate this information, and coordinate responses to maintain homeostasis.

## 2. The Classifications and Functions of Diverse Neuronal and Non-Neuronal Cell Types in the LHA

### 2.1. Neuronal Cell Types in the LHA

It has been recognized for a long time that LHA^Vgat^, MCH, and orexin are the three most fundamental types of neurons located in the LHA region, and their functions involved in feeding regulation and reward and motivation behaviors have been studied separately. However, combined with scRNA sequencing and EASI-FISH technology, a more detailed and systematic classification of the LHA region was established [13,14]. The spatial-molecular organization of the LHA has been fully revealed, with more than 30 subtypes of LHA cells identified recently. Among them, neurons account for more than 50% (about 55%) of the total cell population, while the rest are glial cells. Regarding the transmitter types, the LHA neurons were roughly divided into two main groups—inhibitory and excitatory neurons. The primary inhibitory neuronal LHA population comprises those neurons with expression of the vesicular GABA transporter, VGAT (encoded by *Slc32a1*; LHA^Vgat^ neurons), or glutamic acid decarboxylase (encoded by *Gad1* or *Gad2*), which synthesize and release the neurotransmitter GABA. In contrast, the excitatory LHA neuronal population comprises neurons with the expression of the vesicular glutamate transporter type 2 (VGLUT2, encoded by *Slc17a6*; LHA^Vglut2^ neurons) [15]. Further dimensional reduction (tSNE) classifies two groups of neurons into approximately 20 subgroups with corresponding dominant gene markers [14]. Remarkably, most subtypes of the LHA neurons are either GABAergic or glutamatergic; only a very tiny proportion of them are suspiciously defined as “*Slc17a6*^+^/*Slc32a1*^+^”without acknowledged explanation. The primary identified subgroups of LHA^Vgat^ neurons comprise *Cart*, *Crh*, *Th*, *Lepr*, *Mc3r* and *Gal*. It is reported that *Lepr* and *Nts* simultaneously existed in one subgroup, making their role in homeostasis regulation more intricate [16]. The major identified subgroups of LHA^Vglut2^ neurons consist of *Pmch*, *Hcrt*, *Pdyn*, *Trh*, *Pvalb* and *Pax6*. Certain LHA^Sst^ and LHA^Nts^ neurons are also *Slc17a6*^+^, while a tiny fraction of MCH neurons are *Slc32a1*^+^. Collectively, LHA neurons can be divided into two distinct categories according to the types of neurotransmitters they can release (GABA or glutamate) and can be further divided into several subclasses according to the diversity of neuropeptides. Single-cell sequencing technology and its application in the LHA brain region undoubtedly provides a brand new perspective for the study of the LHA, one that is more comprehensive, detailed, and complete. Some phenotypes that seem to be contradictory when considering the bulk activation or inhibition of large group of neurons may be explained when delving into the molecular subtypes.

Interestingly, neurons within the LHA often form complementary functional pairs that regulate overlapping behavioral processes. Examples include LHA^Vglut2^ and LHA^Vgat^ neurons, which exert opposing excitatory and inhibitory effects; orexin and MCH neurons, which modulate arousal and feeding in distinct ways; and LHA^Lepr^ and LHA^Nts^ neurons, which respond differently to metabolic signals. This molecular and functional diversity contributes to the stability and adaptability of LHA-mediated behaviors.

#### 2.1.1. LHA^Vglut2^ and LHA^Vgat^ Neurons

LHA^Vglut2^ and LHA^Vgat^ neurons are two large groups and their functions in feeding regulation have been discussed for years. In general, they are exactly opposite in terms of feeding regulation. It is worth noting that orexin and MCH neurons both express *Slc17a6* but their effects on feeding are not totally aligned with general LHA^Vglut2^; they are even opposite [15]. Moreover, based on the neuropeptides and hormones they secrete, orexin peptide and MCH, they possess additional complex functions besides feeding, which we will discuss further later. Acute photoactivation of LHA^Vglut2^ neurons suppresses feeding and drives aversion, while genetic ablation potentiates food intake and weight gain under a high fat diet (HFD) but not under a normal chow diet [17,18]. However, the brake effect of excitatory LHA^Vglut2^ on feeding was significantly blunted after HFD feeding. Rossi et al. applied single-cell RNA sequencing and revealed that LHA^Vglut2^ neurons exhibited the greatest proportion of changed genes after 12 weeks HFD feeding. Calcium imaging of LHA^Vglut2^ during sucrose consumption suggested that compared to control diet mice, HFD mice were shown to be progressively less responsive to sucrose consumption, which could be further proven by reduced excitability in patch clamp electrophysiology [19].

Acute photoactivation of LHA^Vgat^ facilitates appetitive, feeding, and reward-related behaviors, while their genetic ablation leads to a markable reduction in these responses [20]. Given that broad manipulation of GABAergic neurons influences a wide range of behaviors, and due to the substantial heterogeneity within this population, it is crucial to investigate the activity dynamics of specific GABAergic subtypes to better understand their distinct functional roles. By implanting microenda oscope with a detachable miniaturized fluorescence microscope, Jennings et al. [20] successfully visualized Ca^2+^ signals from large population of LHA^Vgat^ neurons and revealed that appetitive and consummatory behaviors were encoded and regulated by different subsets of LHA^Vgat^ neurons with barely no overlapping. In addition, it has been reported that LHA^Vgat^ co-expressing *galanin* can mediate food seeking [21]. These observations seem to be consistent with the single-cell sequencing results indicating that diverse molecular subtypes within LHA^Vgat^ groups contribute to the comprehensive effect.

#### 2.1.2. LHA^Lepr^ Neurons and LHA^Nts^ Neurons

LHA^Lepr^ is another subset of GABAergic neurons. Recently, increasing research started to focus on and delve into this small proportion of tissue inside the LHA. Using deep brain cell-type-specific calcium imaging technology, Petzold et al. [22] discriminated a subset of LHA^Lepr^ food-elicited responsive neurons (53%), and the size of recruited LHA^Lepr^ increased with food consumption under acute food deprivation, indicating the dynamics of LHA^Lepr^ neurons participating in the feeding behavior. Among these food-elicited responsive neurons, there exist food-elicited inhibitory and food-elicited excitatory neurons. What is interesting is that the number of a subpopulation of food-elicited inhibitory neurons, defined as “sensitizing food-inhibited cells”, is increased along the feeding progress, facilitating delayed satiation and feeding. However, the number of food-elicited excitatory neurons is not correlated with food activity. Optogenetics activation of food-elicited excitatory neurons suppresses feeding and promotes satiation under acute food deprivation. Owing to the leptin receptor on the membrane, LHA^Lepr^ was endowed with the capacity to respond to the circulation-originated central-accumulated leptin stimulation, but not all of them were. According to Petzold et al., only those food-responsive cells can be selectively activated or suppressed [22], while internally, this small group of neurons is also highly diverse. By using viral RNA TRAP technology, researchers captured crucial information regarding rare cell types, finding that several transcription factors such as *Hdac5*, *Fosl2* and *Crem* are downregulated after 24 h of food deprivation [16]. Despite the low abundance, LHA^Lepr^ neurons are earning increasing focus thanks to their research and transformation potential.

Another related subset of neurons, LHA^Nts^ neurons, is partially GABAergic and partially overlapped with LHA^Lepr^. Thus, LHA^Nts^ neurons can be classified into at least three groups based on whether they co-express *Lepr* or can be activated by dehydration, respectively; these groups are the Nts^Lepr^, Nts^dehy^and Nts neurons [23]. This finding is particularly intriguing, as leptin, secreted from peripheral adipose tissue, serves as a signal of nutritional status, while neurotensin, which is primarily distributed locally, reflects the body’s hydration state. The overlapping expression of LHA^Lepr^ and LHA^Nts^ neurons in the LHA suggests that this region integrates signals related to both feeding and drinking, coordinating behaviors based on internal energy balance and external environmental cues. This aligns with classical views from the last century, which proposed that the LHA is not only the “feeding center” but also the “drinking center” [24,25]. Accordingly, the prioritization of competing needs, such as food, water, and social interaction, appears to be the central role of this neuronal population. Several studies have shown that both LHA^Lepr^ and LHA^Nts^ are engaged in tracking food consumption; however, the LHA^Lepr^ neurons progressively reduce food-seeking behaviors despite hunger pressure in favor of social exploration, while the LHA^Nts^ neurons prioritize water intake even under hunger pressure, de-emphasizing social interaction [22]. These findings, together, imply a more complex integrative role of the LHA beyond sensing energy homeostasis.

#### 2.1.3. Orexin and MCH Neurons

Orexin neurons are a subset of LHA^Vglut2^ and co-express the opioid precursor, prodynorphin (*Pdyn*) [13,26]. Most importantly, orexin neurons can release orexin peptides A and B (also known as hypocretin 1 and hypocretin 2), which can significantly induce feeding behavior. Orexin peptides have two receptors, OX1R and OX2R. OX1R has one order of magnitude greater affinity for orexin A over orexin B, whereas OX2R binds both ligands with similar affinities. Orexin receptors have a wide distribution. Particularly, the ventral tegmental area (VTA) and dorsal raphe nuclei (DR) have a dense OX1R and OX2R distribution. Arcuate nuclei (ARC) have more OX2R, while the locus coeruleus (LC) is especially abundant with OX1R [27]. Generally speaking, OX2R is responsive for wakefulness regulation, whereas OX1R is important in food-seeking regulation [28]. The function of orexin peptide in food intake and energy expenditure has been fully understood based on the previous study of orexin administration in certain brain regions [11,29]. Fasting and low glucose levels can effectively activate orexin neurons [30]. Hormones such as ghrelin and neuropeptide Y (NPY) can also activate orexin neurons with the acid of ARC as the first relay station. Additionally, due to the direct energy signals from circulation, which are relatively slow but robust, orexin can also sense and respond to certain rapid but temporary sensory information from the external environment. Using fiber photometry, the researchers found that the activity of orexin is rapidly increased when mice noticed the food, and food contact can rapidly decrease orexin activity within 1 s of returning to an up-state after food contact was stopped [31]. LHA^Vgat^, LHA^Vglut2^, and orexin neurons are all involved in the regulation of feeding, although they function differently. The functions of these three groups in feeding regulation can be metaphorized as the “engine”, the “brake”, and the “check-engine light”. The “engine” drives the car to run with a “check-engine light” to remind of the real-time status, whereas the “brake” can quickly stop the vehicle’s movement regardless of the normality of the engine. The activation of LHA^Vgat^ drives feeding behavior, with orexin neurons negatively responding to all instances of food contact, whereas LHA^Vglut2^ can suppress feeding behavior. The effects of orexin neurons on energy expenditure rely on adipose tissues. Orexin neurons can induce lipolysis in white adipose tissue as well as induce thermogenesis in brown adipose tissue to promote negative energy balance and reduce adiposity [32]. Therefore, chemogenetic activation of orexin neurons can not only induce increased food intake but also increased water intake, locomotion, RER, and glucose levels, implying the comprehensive involvement of orexin neurons in food intake, energy expenditure, and glucose metabolism [33]. In terms of autonomic actions, orexin neurons can activate the sympathetic system to drive glucose release from liver and glucose uptake by muscle and promote brown-adipose-tissue thermogenesis [34]. However, the fact that the activation of orexin neurons can increase feeding and locomotion is pretty thought provoking, since these two choices seem to be alternatives and normally will not happen to one person simultaneously in the real world. Based on this concern, Tesmer et al. sophisticatedly designed an eight-arm maze that contains almost all the rodent-preferred options including a running wheel, a novel object, a novel mouse, a chow diet, highly palatable food, and so on, to evaluate the choice-making mechanism of mice and the role of the orexin system during this progress. They found that orexin neurons provide the ability for mice to resist the temptation of highly palatable food and choose voluntary exercise, a very valuable ability for humans to maintain their health status when facing increased food temptation [35].

**Figure 1:**
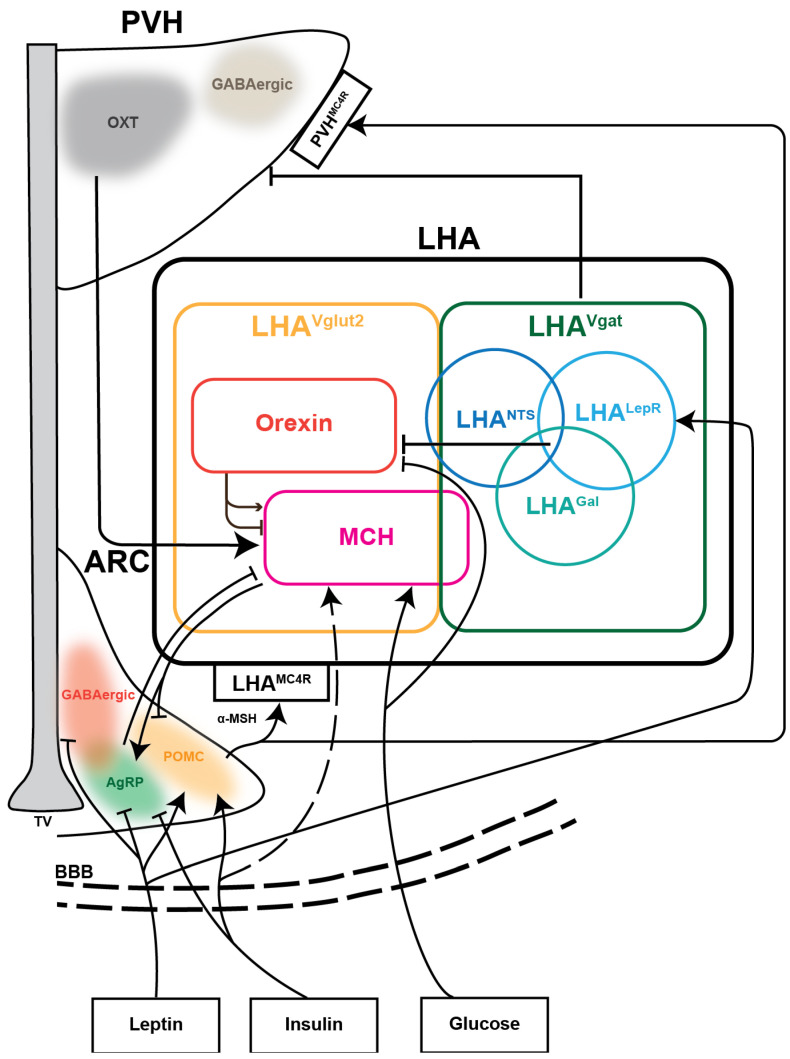
Diagram of different cell types in LHA and the energy signals perception. LHA neurons have two broad classes, which are defined by the expression of GABAergic (LHA^Vgat^) and glutamatergic (LHA^Vglut2^) marker genes. Orexin and MCH neurons are two dominant cell types of LHA^Vglut2^. LHA^Lepr^, LHA^Nts^, and LHA^Gal^ are three dominant cell types of LHA^Vgat^ that have overlapping expression. Neurons in ARC can directly sense the hormonal signals like leptin, insulin, and glucose concentration. Leptin and insulin activate POMC neurons and inhibit AgRP neurons. Leptin can also directly activate LHA^LepR^, which further inhibits orexin neurons. Glucose can activate MCH neurons while inhibiting orexin neurons. Orexin peptides can both excite and inhibit two sub-populations of MCH neurons. AgRP inhibits MCH neurons, while MCH neurons can conversely activate AgRP neurons. POMC neurons activate LHA neurons through MC4R while MCH neurons can reversely inhibit POMC neurons. POMC neurons also activated PVH^Mc4r^. PVH OXT neurons activated MCH neurons and LHA^Vgat^ can inhibit PVH neurons. Abbreviations: BBB, blood–brain barrier; TV, third ventricle; AgRP, agouti-related peptide; POMC, pro-opiomelanocortin; GABA, gamma-aminobutyric acid; ARC, arcuate nucleus; LHA, lateral hypothalamic area; MCH, melanin-concentrating hormone; Lepr, leptin receptor; Nts, neurotensin; Gal, galanin; Mc4r, melanocortin 4 receptor; PVH, paraventricular nucleus; OXT, oxytocin.

As another vital neuropeptidergic neuron in the LHA, MCH neurons can secrete melanin-concentrating hormone, a polypeptide that has important roles in the brain, including the control of energy homeostasis, sleep, learning, memory, and social interactions. Fasting can significantly increase the mRNA expression of MCH. Local administration (ICV) of MCH peptides into the lateral ventricle caused an acute and rapid increase in feeding, while chronic infusion of MCH peptides can induce hyperphagia and obesity in mice [36,37]. However, MCH neurons are neither sufficient nor required for acute appetite in the context of a chow diet [36]. Chemogenetic activation of MCH neurons has no significant effect on short-term food intake but can increase the appetitive responses to food-predictive cues. The physiological concentration of Ca^2+^ in MCH neurons increased with appetitive behavior and during the feeding progress, a characteristic opposite to that observed in orexin neurons [38]. As for the capacity of the glucose response, MCH neurons, similar to orexin neurons, are glucose sensitive. Using electrophysiology, the firing properties upon glucose stimulation of these two types of neurons have been identified. Elevating glucose from 0.2 to 5 mM induced hyperpolarization and suppressed both spontaneous and evoked firing in orexin neurons while depolarizing MCH neurons [30]. Chemogenetic activation of MCH neurons did not cause significant changes in glucose tolerance or insulin sensitivity, while acute chemogenetic inhibition of MCH neurons improves both glucose tolerance and insulin sensitivity, suggesting that the inhibition of MCH neurons benefits whole-body glucose metabolism [39]. Besides glucose sensing, MCH neurons also have a complete opposite function in locomotion regulation and energy expenditure compared to orexin neurons. The activation of MCH neurons inhibits locomotion and promotes fat accumulation. The knockout of MCH neurons causes increased energy expenditure, hyperactivity, and food-seeking behaviors [40]. A recentl study suggested that MCH neurons might regulate brown adipose tissue (BAT) activity and energy expenditure through long projections to the medullary raphe nucleus (MRN) to inhibit sympathetic input to BAT [41]. The integrated neuron subtypes are summarized in Figure 1.

### 2.2. Non-Neurons in the LHA

In addition to neurons, glial cells also critically contribute to the function of the LHA, interacting closely with neurons and participating in the regulation of energy sensing and obesity pathogenesis. Astrocytes play an active role in the brain by expressing various receptors for neurotransmitters and releasing various transmitters and neuroactive molecules, such as GABA, glutamate, ATP, adenosine, and D-serine. Importantly, astrocytes are highly involved in brain glucose utilization. When sensing that neuronal activity is increased, astrocytes will uptake more glucose for glycolysis and produce lactate in the extracellular environment of neurons nearby that can be imported into neurons through monocarboxylate transporters (MCTs) and oxidized to ATP to supply the energy demand of neurons. This famous model is called astrocyte–neuron lactate shuttle (ANLS) [42]. Astrocyte-produced lactate is always considered as a critical energy substrate, combined with glucose, as the energy transfer agent that connects neurons and astrocytes. A recent study specifically emphasized its role as a regulator of the orexin system. By conditional knockdown of MCT2 or MCT4, Alice et al. found that lactate is necessary for sustaining orexinergic activity and consolidating wakefulness through MCT4 (monocarboxylate transporters 4) in astrocytes and MCT2 in orexin neurons [43]. To further clarify the content of ANLS, researchers revealed recently that the GLUT1 on the membrane of astrocytes is a potential genetic target for energetic diseases such as obesity or neurodegeneration since partial reduction of GLUT1 of astrocytes can significantly enhance brain glucose metabolism and relieve HFD-induced glucose resistance [43,44]. Microglia cells are critical contributors to HFD-induced obesity pathogenesis and have a close interaction with astrocytes. Microglia cells are extremely responsive in the inflammatory progress, which is triggered by excessive saturated fatty acids (SFAs) from the diet. By releasing pro-inflammatory cytokines, chemokines, and reactive oxygen species (ROS), glia cells are quick responders to excessive energy signals [43]. Astrocytes and microglia together release multiple factors that alter the function and metabolic activity of each other and other CNS cells, including small molecules/ions, cytokines, and an array of lipids, metabolites, and other factors in isolation or packaged into lipo-particles and other vesicles [45]. Owing to the wide distribution of glia cells in the CNS and their general effects, without clear discrimination between hypothalamus regions yet, more detailed information about glia cells in hypothalamus will be omitted here.

## 3. The LHA as the Intermediate Region for Feeding and Rewarding

The LHA, as an intermediate region, locationally or functionally, receives the signals and messages from peripheral organs transmitted by the axon terminal from the ARC and provides feedback information to them. It also forms a direct or indirect connection to the monoaminergic system in the VTA and nucleus accumbens (NAc) for feeding-related rewarding regulation. In addition, as one of the many intermediate regions, the LHA can also interconnect with other regions, allowing the nutritional signals or feeding-related stimuli to be further executed and buffered for full reaction [46]. Here, we will elucidate the step of peripheral energy-related messages and explain how these signals are recognized by the LHA, transformed, and eventually turned into complex behaviors.

### 3.1. The LHA and Energy Signals Perception

The brain-sensing model has been reported to conform to the parallel/distributed model, with diverse stimuli sensed in parallel by several brain sensors and distributed throughout the brain circuits [47]. Among the stimuli that reflect the energy status of the body are circulating hormones, such as leptin, insulin, and ghrelin. They are produced from their organs of origin, enter the circulation system, and, finally, reach the very crucial destination, the central nervous system (CNS), which they have access to through either the blood–brain barrier (BBB) or the median eminence (ME). Glucose is another unique stimulus because of its dual identity, simultaneously functioning as an energy substance and an information transmitter. ARC neurons are indispensable components of the brain sensors as the first-order neurons of these stimuli, possessing abundant hormonal receptors and glucose-responsive properties. LHA neurons, on the other hand, function as more than just second-order neurons thanks to the hormonal receptors on the membrane and their glucose-responsive properties as well as the complex connections among the ARC, PVN, VMH, and LHA. In this part, we will emphasize the direct sensing of LHA neurons and message exchange between LHA neurons and other brain sensors.

#### 3.1.1. LHA Neurons Sense Feeding-Related Signals

Feeding-related hormones, including anorexigenic hormones (e.g., leptin and insulin) and orexigenic hormones (e.g., ghrelin), dynamically regulate energy homeostasis through hypothalamic circuits. In the previous section, we introduced an important subtype of LHA neurons, LHA^Lepr^, which are capable of sensing and responding to the central leptin signals independently of other pathways. Activation of LHA^Lepr^ neurons through the central administration of leptin into the LHA is sufficient to suppress food intake and reduce weight gain [48]. Besides the LHA^Lepr^, other LHA neuronal populations also respond to leptin signaling. Notably, MCH neuron is one of the key-acting downstream of leptin. In *ob/ob* leptin-deficient mice, the MCH mRNA levels are increased and can be rescued through the administration of leptin [49]. Moreover, the genetic deletion of MCH in *ob/ob* mice attenuates the obesity phenotype, primarily by enhancing energy expenditure, suggesting that leptin exerts an inhibitory role on MCH neurons [48,50].

Orexin neurons can be modulated by leptin, albeit indirectly through the LHA^Lepr^ [51]. Instead, leptin activate a subset of galanin-expressing LHA^Lepr^ (LHA^Lepr/Gal^), which, in turn, inhibits the downstream orexin neurons through galanin release rather than GABAergic transmission [52,53]. By contrast, this direct local inhibitory innervation from LHA^Lepr^ to orexin is absent between LHA^Lepr^ and MCH neurons [54]. However, it is hypothesized that a polysynaptic circuit may link LHA^Lepr^ neurons to MCH neurons potentially via excitatory input from nearby orexin neurons [55,56].

LHA neurons also respond directly to insulin as well. In vitro electrophysiology recordings show that insulin can increase the excitability of a subset of MCH neurons via phosphoinositide 3-kinase (PI3K) signaling. Although insulin signaling in MCH neurons appears dispensable under normal physiological conditions, it becomes functionally significant in the context of obesity. In obese mice, insulin enhances MCH neuron activity, contributing to hallmark features of obesity such as reduced locomotor activity and insulin and glucose resistance [57].

The stomach-derived acylated peptide ghrelin is recognized as the most potent known orexigenic hormone. ARC, VMH, and DMH have a more dominant ghrelin receptor distribution, while LHA neurons still have ghrelin receptors, called GHSR. Intra-LHA ghrelin injection induced a transient increase in food intake with free access to food and a transient increase in locomotion without access to food. GHSR did not co-localize with orexin, MCH, or Gad2; instead, most GHSR neurons are *nNOS* or *Nts* [58].

#### 3.1.2. LHA Neurons Sense the Nutrients Signals

Glucose is the basic energy source of the whole body, especially the central nervous system, which consumes 10% of the blood glucose. In addition, it also represents signals and transmits energy information from time to time. The basis of the CNS’s role in sensing the fluctuation in glucose is due to glucose-sensing neurons, more specifically, glucose inhibitory (GI) and glucose excitatory (GE) neurons. Glucose inhibits specific populations (19%) including orexin neurons in the lateral hypothalamus, while conversely activating other cell groups (38%) such as lateral hypothalamic MCH neurons [59]. Physiologically relevant concentrations of glucose directly inhibit the firing of orexin neurons by triggering hyperpolarization and decreasing membrane resistance [30]. This rough negative correlation between orexin neurons and glucose concentration has been established for decades, while the exact messages tracked by orexin neurons have not been clear until recently. Viskaitis et al. designed and implemented an experimental paradigm that combined telemetry of carotid electrochemical glucose sensors and fiber photometry to record the neural activity of orexin neurons while monitoring the glucose concentration. They found that the population of orexin neurons is rapidly inhibited by an increase in blood glucose, and this inhibition tracks the rate of change in blood glucose [60]. The mechanism of MCH neurons sensing glucose is through the ATP-mediated closure of Sur1-containing K_ATP_ channels and is negatively regulated by UCP2. To date, the precise relationship between MCH neurons and glucose homeostasis remains unclear. In addition to MCH and orexin, another less commonly discussed LHA neuronal subtype, that expressing neuropeptide Y (NPY), also responds to glucose levels, albeit through a distinct mechanism. The LHA^NPY^ neurons can be activated in response to hypoglycemia induced by insulin administration or acute food deprivation [61].

In addition to being the glucose sensing region, the LHA is also the center for amino acid homeostasis. Compared to glucose, the composition of animo acid is more complicated. There are 20 kinds of L-animo acid ingested from dietary protein, with 9 of them being essential animo acids. This sensing occurs through vagal afferent inputs into the brain. Lysine (Lys) is one of the most studied essential animo acids and is highly related to the LHA. Intragastric intubation of lysine solution in Lys-deficient rats causes a great response in a wide area of the brain, especially in the LHA. However, there were no neurons that responded directly to Lys application to single neurons in the LHA, indicating that neurons in the LHA exhibit neuronal plasticity, responding to a previously neutral stimulus that now signals the presence of Lys solution availability [62].

#### 3.1.3. Integration of Neuronal Circuits Within Hypothalamus Brain Sensors

Besides their direct sensing capabilities, LHA neurons, together with other hypothalamic regions such as the ARC, DMH, VMH, PVN, and ME, constitute an integrated central energy-sensing network. The neuronal circuits connecting the LHA with these regions are largely bidirectional, implying that LHA neurons are not only passive recipients of metabolic signals but also actively modulate incoming information [63].

The ARC serves as a primary relay station and contains three major clusters of neuronal populations—POMC, AgRP, and non-AgRP GABAergic neurons. This region is highly enriched with leptin and insulin receptors. AgRP neurons, which are GABAergic, are inhibited by leptin and play a key role in stimulating appetite. While when AgRP neurons are activated under a negative energy balance, they secrete AgRP and NPY, which can inhibit MCH neurons in the LHA [56]. Non-AgRP GABAergic neurons, the principal executors of leptin signaling, are suppressed by leptin and, thereby, disinhibit the downstream POMC neurons. Leptin also directly binds to leptin receptors on POMC neurons, decreases its IPSCs, and promotes the secretion of alpha-melanocyte-stimulating hormone (αMSH), which next activates melanocortin receptors (MC4R and MC3R) located on the membrane of the LHA and PVN to reduce food intake [64,65]. It is worth noting that besides receiving input from the leptin-responsive ARC, LHA^Mc4r^ can directly influence glucose metabolism through sympathetic nerve activity by modulating sympathetic nerve activity to interscapular brown adipose tissue (iBAT) [66].

Conversely, LHA neurons, including MCH and orexin neurons, send dense projections back to the ARC. MCH neurons stimulate the release of NPY and AgRP while inhibiting the release of CART and αMSH [67]. Similarly, orexin neurons can excite NPY neurons in the ARC, facilitating the release of NPY for feeding behavior [68]. The PVH and LHA maintain robust bidirectional connectivity. MCH and orexin neurons project densely to the PVH [27,69], while vasopressin (AVP) and oxytocin (OXT) neurons in the PVN provide excitatory input to MCH neurons, depolarizing their membrane potential and increasing spike frequency [70]. Additionally, LHA^Vgat^ project extensively to the PVH, while optogenetic stimulation of LHA^Vgat^ → PVN fibers evokes monosynaptic IPSCs in PVH neurons and significantly enhances feeding behavior in a GABA-dependent manner [71].

Furthermore, MCH neurons have been shown to provide dense projections to the median eminence (ME). Their activation increases micro-vessel fenestration, thereby enhancing ME barrier permeability and facilitating leptin access to the ARC [72].

#### 3.1.4. Summary of the Peripheral Signaling Sensing

Hormone levels serve as critical indicators of the body’s overall energy state. ARC neurons, as the primary messengers for energy sensing, can be activated or inhibited by various hormones signals. These signals are then transmitted to the downstream regions, including the lateral hypothalamic area (LHA), which acts as a secondary messenger in this regulatory pathway. In turn, neurons in the LHA densely project back to the ARC, inhibiting POMC neurons and activating NPY neurons. This bidirectional communication forms hypothalamic microcircuits that involve positive feedback and negative feedback mechanisms [65,66,68,69]. Additionally, LHA neurons can themselves directly sense hormone signals as well, and they play essential functional roles. The disruption of this signal cascade can severely impair whole-body energy homeostasis, as accurate hormonal perception forms the fundamental basis for appropriate behavioral responses. The peripheral signaling sensing of LHA neurons are summarized in Figure 1.

### 3.2. LHA and Other Intermediate Region Circuits

#### 3.2.1. Neuronal Circuit Within the LHA

The heterogeneous molecular subtypes of the LHA neurons shape complex intra-LHA neuronal circuits that are complementary and functionally stable. Considering the opposing roles of orexin and MCH neurons in feeding behavior and sleep regulation, it is interesting to determine the interaction between these two groups. Artificially applied orexin-A peptides can only excite a minor part MCH neurons; the rest were either unaffected or inhibited [73]. Consistently, the application of orexin-B peptides on MCH neurons induces both excitatory and inhibitory responses during application, indicating the sub-population of MCH neurons. After the ablation of OX2R, the receptor of orexin-B, Shuntaro et al. found impaired insulin sensitivity and reduced transitions from NREMS to REM, indicating the inhibition of metabolism-regulating MCH neurons and the excitation of sleep-regulating MCH neurons, respectively [74]. Besides direct interaction through orexin peptides, orexin neurons can inhibit MCH neurons via GABAergic interneurons [73]. Except for MCH neurons, orexin neurons can activate another subtype of LHA neurons, that is, LHA^Gad65^ neurons. The role of orexin neurons in promoting voluntary locomotion and physical activity has already been studied, while Kosse et al., surprisingly, found that orexin can rapidly recruit and activate local LHA^Gad65^ neurons that are locomotion-associated because of the pre-increased activity before locomotion onset and the lasting activity during locomotion [75]. Additionally, orexin neurons can receive GABA projections from LHA^Lepr^. They innervate a subset of orexin neurons and form inhibitory synaptic connections that further project to the PVN by releasing orexin peptide. Since the PVN is directly responsible for HPA axis activity, this circuit can integrate metabolic state and stress responses through functional connections among LHA^Lepr^ neurons, orexin neurons, and the HPA axis. Under a negative energy balance with low leptin, more orexin is released and, thus, HPA axis activity and corticosterone release are increased. Conversely, high leptin levels suppress orexin-mediated HPA axis responses and corticosterone release [76].

#### 3.2.2. Circuit of the LHA, Extended Amygdala, and PBN

**Figure 2:**
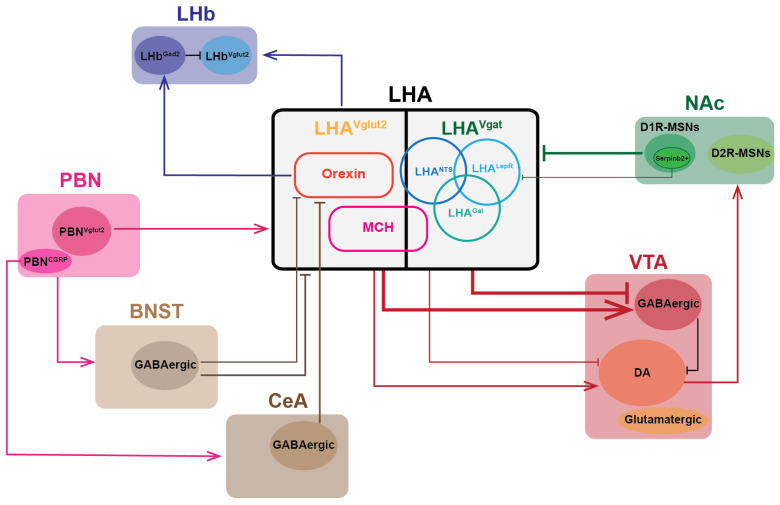
Summary of connections between LHA and other brain regions’ neurons implicated in energy regulation. Abbreviations: BNST, bed nucleus of the stria terminalis; LHb, lateral habenula; MCH, melanin-concentrating hormone; Lepr, leptin receptor; Nts, neurotensin; Gal, galanin; NAc, nucleus accumbens; PBN, parabrachial nucleus; VTA, ventral tegmental area; CeA: central amygdala; DA: dopamine; D2R-MSNs: dopamine receptor 2-expressing medium spiny neurons; D1R-MSNs: dopamine receptor 1-expressing medium spiny neurons; CGRP, calcitonin gene-related peptide.

The extended amygdala comprises two components that are involved in both reward and feeding, which are the bed nucleus of the stria terminalis (BNST) and the central amygdala (CeA). BNST GABAergic neurons project densely to LHA^Vglut2^ but not LHA^Vgat^. Optogenetic activation of BNST GABAergic neurons → LHA^Vglut2^ induces feeding while optical inhibition suppresses food intake [18]. Parabrachial nucleus (PBN) neurons simultaneously send projections to the LHA, CeA, and BNST, with almost no overlapping of PBN neurons. For LHA, LHA^Vglut2^ is the main target of PBN, and the optogenetic activation of PBN^Vglut2^ → LHA^Vglut2^ resulted in a reduced total food intake under the fast-refeeding paradigm owing to the reduced motivation to obtain food [77]. The same effect of reduced feeding exists with the activation of a subset of PBN^CGRP^ (CGRP, calcitonin gene-related peptide) neurons in the CeA, but with an entirely different mechanism. The activation of PBN^CGRP^ → CeA induced aversion-caused anorexia to reduce feeding, while the activation of PBN^Vglut2^ → LHA^Vglut2^ reduced the motivation for eating [78,79]. Notably, PBN^CGRP^ neurons received inhibitory input directly from AgRP neurons to reduce PBN^CGRP^ → CeA induced anorexia and delay satiety, while the LHA^Mc4r^ neurons receive αMSHsecreted from POMC to facilitate feeding behavior. LHA^Vglut2^ neurons received monosynaptic input from both the PBN and BNST and the effect on feeding depends on the food suppression effect of LHA^Vglut2^ we mentioned above. Except for LHA^Vglut2^, orexin neurons can be excited by input from the BNST and CeA. Disinhibition of the CeA or BNST using microinjections of a GABA_A_ receptor antagonist increased *Fos* immunoreactivity in orexin neurons and induced cardiorespiratory excitation in wild-type mice but not in mice lacking orexin neurons [80]. The neuronal circuits between LHA and other intermediate regions are summarized in Figure 2.

#### 3.2.3. LHA and LHb

The lateral habenula (LHb) has long been considered to promote negative emotional stages through the indirect inhibition of midbrain dopamine systems and its dysfunction is highly associated with mood disorders [81]. LHA^Vglut2^ neurons have dense projections to the LHb, mediating aversive signals. Optogenetic activation of the LHA^Vglut2^ → LHb circuit induces a strong aversive response in real-time-place-preference assay, causing obvious negative valence and rapid prediction of the upcoming negative event [82]. Orexin neurons, on the other hand, subset of LHA^Vglut2^, also send projections to the LHb. These include, more specifically, LHb^Gad2^ neurons, which function through the neuropeptide orexin and the orexin receptor (OX2R). LHb neurons used to be seen as exclusively glutamatergic until single-cell sequencing evidence revealed a small population of cells that express *Gad2*. They locally inhibit the nearby LHb^Vglut2^ neurons and receive the orexin projections through orexin peptide to regulate aggression behavior. Optogenetic activation of the orexin → LHb circuit can promote aggression and aggression conditioned place preference (CPP), extending the role of orexin neurons in the social area [83].

### 3.3. LHA and Monoaminergic System

#### 3.3.1. LHA and VTA

The neuronal circuit from the LHA to VTA is pretty important for motivation and rewarding behaviors. Both GABAergic and glutamatergic neurons send projections to the VTA, but through different patterns. The components of the VTA region are relatively clear, with 65% dopamine (DA) neurons, 35% putative GABAergic neurons, and 5% glutamatergic neurons [84]. Although the monosynaptic input from the LHA to VTA DA neurons has been confirmed to have more glutamatergic input than GABAergic input, there is controversy surrounding the contribution of this monosynaptic input [85]. Instead, compared to VTA DA neurons, putative GABAergic neurons received greater input from LHA no matter the glutamatergic input of GABAergic. But these two parallel circuits possess entirely distinct performances in feeding and other motivational behaviors. LHA^Vgat^ neurons send inhibitory projections to VTA GABAergic neurons, which form an inhibitory circuit locally with VTA DA neurons. Thus, the photo-activation of the LHA^Vgat^ → VTA GABAergic neurons will disinhibit VTA DA neurons, facilitating the release of dopamine to downstream regions including NAc. Not merely limited to feeding, the activation of LHA^Vgat^ → VTA GABAergic neurons promote approach behavior as well as social interaction, as it is characterized by the investigation of the most proximal salient object, no matter whether that is food or a novel object. Conversely, the activation of LHA^Vglut2^ → VTA shows an entirely different phenotype, demonstrating reduced feeding and social interaction and avoidance [86]. LHA^Lepr^ is a subset of GABAergic neurons, which follow the same projection pattern as LHA^Vgat^ to the VTA [48]. Consistent with LHA^Vgat^, LHA^Lepr^ make inhibitory projections to the VTA, mainly to VTA GABAergic neurons, and the photo-stimulation of the LHA^Lepr^ terminal increased the motivation for feeding. Additionally, water reinforcement behavior was observed after the activation of this circuit, owing to the co-expression of *Nts* in LHA^Lepr^ with a percentage of approximately 68% [87]. In summary, the LHA^Vgat^ → VTA GABAergic neurons circuit promotes a general motivation for investigation rather than being fixed to a particular behavior, reflecting that the general level of regulation is gradually increased [46].

Additionally, MCH and orexin neurons are also involved in the circuit system of LHA → VTA. Both orexin and MCH receptors are expressed in the VTA. Using extracellular and whole-cell patch-clamp recording techniques, orexin peptides were found to excite the VTA GABAergic and DA neurons through the expression of orexin receptors in the VTA, while MCH peptides can exert both the facilitation and inhibition of action-potential firing, suggesting that VTA-projecting orexin and MCH neurons can directly modulate DA neuron activity [88,89].

#### 3.3.2. LHA and NAc

Downstream of the VTA and upstream of the LHA, the nucleus accumbens (NAc) links the signals transmitted from and feedback to LHA^Vgat^. Nucleus accumbens shell (NAcSh) projections to LHA are implicated in mediating such feeding control. Two dominant neuron subtypes exist in the NAcSh, dopamine receptor 1-expressing medium spiny neurons (D1R-MSNs) and dopamine receptor 2-expressing medium spiny neurons (D2R-MSNs). The former have been shown to make dominant inhibitory projections to LHA^Vgat^ neurons (whose activation is appetitive) and have a role in the disinhibition of feeding behavior [90]. Generally speaking, the activity of the NAcSh^D1R-MSNs^ → LHA^Vgat^ pathway is inhibited during the eating period under a normal chow diet. Specially, when mice were in the condition of favoring eating, such as during food restriction or in the context of a high-fat diet, the NAcSh^D1R-MSNs^ → LHA^Vgat^ synapse underwent robust inhibitory LTD to facilitate enhanced and prolonged eating behavior even without energy deficit, contributing to weight gain and even obesity [91]. Optogenetic activation of the NAcSh^D1R-MSNs^ → LHA^Vgat^ pathway can rapidly stop mice from eating even under hunger. Conversely, mice will keep eating despite enough energy sources when this pathway is inhibited. Recently, more precise work focusing on the molecular subtype of NAcSh^D1R-MSNs^ → LHA has been done. By applying single-cell RNA sequencing as well as spatial transcriptome techniques combined with single-molecule FISH (smFISH) validation, the author successfully identified a cluster of D1R-MSNs^Serpinb2+^ neurons that form inhibitory projections to LHA^Lepr^ neurons. Opposite to the original results, the activation of the NAcSh^D1R-MSNs^ → LHA^Lepr^ neuronal circuit improves feeding behavior and can overcome leptin-mediated feeding suppression. Owing to the heterogeneity of D1R-MSNs neurons, *serpinb*^2+^ neurons and *serpinb*^2−^ neurons have opposite roles in regulating feeding [92]. Except for LHA^Vgat^ neurons, LHA^Vglut2^ neurons have also been found to be involved in the NAcSh projections; still, more research is need to further unravel the mystery. Collectively, NAcSh → LHA projection functions as a switcher to help rapidly transform between different behavioral states in response to changing external conditions.

LHA^Vgat^ also received indirect input from the NAc by mediating PVT neurons. This long-projection circuit primarily encodes sensory information related to licking and reward consumption, contributing to the multiplexed signal guiding reward-seeking behavior [93].

## 4. Summary

As the second messenger of energy signals and the intermediate region for feeding and reward, the LHA can not only perceive signals from the peripheral environment through hormones but also function largely as the executor by sending wide-range projections within and outside the hypothalamus. Under a normal state, low circulating glucose levels and high ghrelin levels indicate the state of hunger. This will activate the AgRP and GABAergic neurons in the ARC and inhibit the POMC neurons, facilitating feeding and suppressing satiety. The activated AgRP and GABAergic neurons send inhibitory projections to the nearby POMC, aligning with the effect of hormones. Low glucose levels and high ghrelin levels activate the orexin neurons and promote the secretion of orexin peptide, further activating NPY neurons in the ARC through OX1R. Depending on the direct projection AgRP → PBN^Vglut2^, which is inhibitory, the activation of AgRP can suppress the activation of PBN^Vglut2^ → LHA^Vglut2^ and promote feeding. Meanwhile, input from the NAc to LHA^Vgat^ promotes food-seeking behavior and, of course, the increased locomotion as a price for it. During the process of food intake, the activity of LHA^Vglut2^ and orexin increase gradually, reflecting the real-time energy state. When the organism is no longer in a state of negative energy balance, the effect of suppression will exceed the promotion and induce feeding termination. Meanwhile, POMC neurons will be activated and release αMSH. LHA^Mc4r^ will recognize this signal and promote satiety. While under an obesity situation or HFD feeding for more than 8 weeks, this system undergoes great changes. An increased amount of saturated fatty acids (FAs) from the periphery crosses the BBB and induces an inflammatory response through microglia in hypothalamic neurons. Hormones receptors such as insulin, leptin, and ghrelin receptors lose their sensitivity and develop whole-body glucose and insulin resistance. Additionally, the real-time peripheral messages cannot be correctly transmitted into the CNS. Mistakes start to occur. The activity of POMC and LHA^Vglut2^ is continuously weakened, causing eating behavior that is difficult to stop. In addition, the feeding-related reward phenotype mediated by the circuit among the LHA, VTA, and NAc changed a lot. Adaptive changes in the expression of DA neurons, D1R and D2R, due to repeated HFD-mediated activation of D1R signal transduction, led to downregulated D1R and D2R availability. The NAc → LHA satiety circuit may be inhibited due to HFD-induced disruption of D1R signalling.

## 5. Concluding Remarks and Future Perspectives

Research on the LHA has a long history, yet many fundamental questions remain unresolved. One major challenge arises from the paradoxical relationship between simplicity and complexity. In recent years, single-cell sequencing technologies have allowed neuroscientists to gain unprecedented insight into the molecular diversity of LHA neuronal subtypes. However, this technological breakthrough has also revealed a staggering level of complexity—not only in the LHA, but across the entire brain—raising the critical question of how to interpret and simplify such intricate data. The abundance of gene markers and overlapping expression profiles can easily obscure the functional relevance of specific cell populations.

This leads to a second key challenge: how to gain a comprehensive perspective of the LHA’s diverse functions and translate that knowledge into meaningful biological and clinical insights. Manipulation tools such as optogenetics and chemogenetics are powerful and effective and can successfully amplify minor changes for better research. Yet, as the research delves deeper into complex systems, it becomes increasingly important to contextualize findings within real-world physiological and behavioral scenarios. Ultimately, the value of neuroscience—particularly in biomedical applications—lies in its relevance to actual biological conditions.

The third challenge concerns the continuous advancement and refinement of the research methodologies. The manipulation methods using optogenetics or chemogenetics once represented the frontier of neural manipulations; they may no longer suffice given the complexity now revealed by molecular profiling. As the veil of the molecular characteristics of many different neuronal subtypes is being revealed, it becomes clear that traditional methods need to evolve. Encouragingly, a growing number of researchers are developing advanced tools that offer improved spatiotemporal resolution and can accommodate the dynamic, multifunctional nature of neural circuits. Continued methodological innovation will be essential to propel the field forward and yield deeper insights into LHA function.

## Data Availability

Not applicable.
